# mRNA-Mediated Gene Supplementation of Toll-Like Receptors as Treatment Strategy for Asthma *In Vivo*

**DOI:** 10.1371/journal.pone.0154001

**Published:** 2016-04-21

**Authors:** Franziska Zeyer, Benedikt Mothes, Clara Will, Melanie Carevic, Jennifer Rottenberger, Bernd Nürnberg, Dominik Hartl, Rupert Handgretinger, Sandra Beer-Hammer, Michael S. D. Kormann

**Affiliations:** 1 Department of Pediatrics I - Pediatric Infectiology and Immunology - Translational Genomics and Gene Therapy, University of Tübingen, Tübingen, Germany; 2 Department of Pharmacology and Experimental Therapy and ICePhA, University of Tübingen, Tübingen, Germany; University of Alabama-Birmingham, UNITED STATES

## Abstract

Asthma is the most common chronic disease in childhood. Although several therapeutic options are currently available to control the symptoms, many drugs have significant side effects and asthma remains an incurable disease. Microbial exposure in early life reduces the risk of asthma and several studies have suggested protective effects of Toll-like receptor (TLR) activation. We showed previously that modified mRNA provides a safe and efficient therapeutic tool for *in vivo* gene supplementation. Since current asthma drugs do not take patient specific immune and TLR backgrounds into consideration, treatment with tailored mRNA could be an attractive approach to account for the patient’s individual asthma phenotype. Therefore, we investigated the effect of a preventative treatment with combinations of *Tlr1*, *Tlr2* and *Tlr6* mRNA in a House Dust Mite-induced mouse model of asthma. We used chemically modified mRNA which is–in contrast to conventional viral vectors–non-integrating and highly efficient in gene transfer. In our study, we found that treatment with either *Tlr1/2* mRNA or *Tlr2/6* mRNA, but not *Tlr2* mRNA alone, resulted in better lung function as well as reduced airway inflammation *in vivo*. The present results point to a potentially protective effect of TLR heterodimers in asthma pathogenesis.

## Introduction

Based on the principles of the hygiene hypothesis, several studies indicate that the onset of atopy and allergic asthma is less frequent in children having been exposed to an environment rich in microbes in their early childhood [1–3]. Increased hygiene standards in Western lifestyle however go along with a reduced contact to microbes, facilitating the development of these diseases and contributing to the rise of atopy in developed countries [4–6].

As primary sensors of the immune system, Toll-like receptors (TLRs) are responsible for recognizing and responding to microbes and microbial components, so-called pathogen-associated molecular patterns (PAMPs). By inducing the secretion of certain “instructive” cytokines, TLRs furthermore influence T-cell development, mainly towards a T helper cell type 1 (Th1) dominant phenotype [7]. PAMPs are involved in the pathogenesis of atopic diseases such as asthma, allergic rhinitis and allergic dermatitis. The initial triggers for these diseases are still not entirely understood. However, in the last years, multiple studies demonstrated that an imbalance of T helper cell responses plays an important role in their development [8,9]. In the case of asthma, the predominance of a Th2 pattern leads to an increased production of chemokines, as well as allergen-specific immunoglobulins, thus causing airway inflammation, eosinophilia and mucus hypersecretion in the lung [10–12]. The clinical presentation of atopic asthma eventually consists of wheezing, airway obstruction, breathlessness and cough, often accompanied by recurrent bronchitis or pneumonia [13,14].

The perspectives of gene therapies in the field of immunology have been of great interest in recent years. DNA-based gene therapy however implies the threat of genomic integration and immunogenicity and is furthermore often limited by low transfection efficiency. The application of nucleotide chemically modified mRNA (cmRNA) however, circumvents these threats and further ensures high stability, thus representing a promising therapeutic tool [15–19]. Previous work by our group and others has shown that delivery of cmRNA leads to therapeutic levels of protein expression as a result of high gene transfer efficiency, higher stability and/or low immunogenicity, and hence, can even be utilized for live-saving genome editing *in vivo* [17,19,20].

We found that polymorphisms in TLR1, 6 and 10, all capable of forming heterodimers with TLR2, have shown protective effects on atopic asthma in humans [21]. These effects were further associated with an increased expression and elevated peripheral blood mononuclear cell secretion of Th1 cytokines. Recent studies suggest a protective role of TLR6 activation in asthma via the regulation of cytokine expression by dendritic cells [22].

Here, based on the data of asthma-protecting TLR-haplotypes, we investigated the intratracheal application of combinations of chemically modified *Tlr1*, *Tlr2* and *Tlr6* mRNA in a House Dust Mite (HDM)-induced mouse model of asthma. We further analyzed how this treatment *in vivo* differentially modulates neutrophilic and eosinophilic airway inflammation and lung function.

## Methods and Materials

### mRNA production

mRNA transcripts of *Tlr1*, *2* and *6* were produced as previously described [17]. In brief, T7-promoter-containing pVAX.A120-vectors encoding for Tlr1, 2 and 6 were linearized and transcribed *in vitro* into chemically modified mRNA, incorporating 25% 2-Thio-UTP and 25% 5-Methyl-CTP (TriLink Bio Technologies) using the T7 MEGAscript kit (Ambion). Modified mRNA was purified using the MEGAclear kit (Ambion) and dissolved in RNase-free DEPC-water.

### Animal experiments

Female BALB/c mice were purchased from Charles River Laboratories at an age of six to eight weeks. Mice were kept under specific pathogen-free conditions and maintained on a 12-h light-dark cycle. Food and water were provided *ad libitum*. All animal experiments were approved by the ethics committee of the regional board of Tübingen and carried out in strict accordance with the recommendations in the Guide for the Care and Use of Laboratory Animals at the University of Tuebingen, the German Law for the Protection of Animals and FELASA regulations. All efforts were made to minimize suffering of the animals.

Intratracheal procedures were carried out using a high-pressure spraying device (PennCentury) under antagonizable anesthesia with a mixture of medetomidine (0.5 mg/kg), midazolam (5 mg/kg) and fentanyl (50 μg/kg). After treatment antidot (atipamezol (50 μg/kg), flumazenil (10 μg/kg) and naloxon (24 μg/kg)) was injected s.c. Per time point, mice received 100 μl DEPC-water containing either 20 μg of the respective *Tlr* mRNA or water as control. HDM (Greer Laboratories) was administered as 100 μg extract dissolved in 100 μl PBS. At experimental endpoints, animals were euthanized using 120 mg/kg Na-Pentobarbital.

### Cell preparations

Lungs were lavaged with 1 ml PBS in order to obtain BALF. Total BAL cells were subsequently centrifuged and analyzed. Differential cell counts were performed by counting at least 100 cells (macrophages, monocytes, neutrophils, eosinophils, basophils and lymphocytes) from different fields of view on cellspin preparations (CellspinII, 6°C, 75 *g*, Ramp 4, Break 6; Tharmac). Lungs were harvested and incubated for 1 hour at 37°C in a digestion solution containing 1 mg/ml collagenase type 1 (Life Technologies), 1 mg/ml Dispase (Corning) and 500 U DNase (EPICENTRE Biotechnologies). Thereafter, digested lungs were passed through a 70 μm cell-strainer in order to receive a single-cell suspension. Erythrocytes were lysed using ACK-Lysing Buffer (Life Technologies) and cells were counted and subsequently subjected to flow cytometry (FACS) analysis.

For FACS, monoclonal anti-mouse antibodies F4/80-Pacific Blue rat IgG2A (final dilution 1:100; AB_893475, clone BM8), CD19-PerCP/Cy5.5 rat IgG2A (final dilution 1:200; AB_2072925, clone 6D5), CD3_Ɛ_ -Brilliant Violet rat IgG2b (final dilution 1:20; clone 17A2, AB_2562555) (all BioLegend), Siglec-F PE rat IgG2A (final dilution 1:100; AB_394341, clone E50-2440), CD11b-PE.Cy7 (final dilution 1:200; AB_2033994, clone M1/70), CD11c-APC/Cy7 hamster IgG1 (final dilution 1:500; AB_10611727, clone HL3) (all BD Pharmingen) and Ly6G-APC rat IgG2b (1:833; AB_469475, clone RB6-8C5) (eBioscience) were used to stain lung cells according to the manufacturer’s instructions.

### Airway resistance

At the predetermined endpoint of the study, airway resistance in response to methacholine (Sigma-Aldrich) was determined using the *ex vivo* model of the IPL as previously described [19, 20]. In short, *in situ* mouse lungs were placed in a thorax chamber and mice were ventilated *via* a tracheal cannula. Ventilation was set to 90 breaths/minute with negative pressure ventilation between -2.8 cm H_2_O and -8.5 cm H_2_O. To prevent atelectasis, a hyperinflation was triggered every 5 min (-25 cm H_2_O). Perfusion of lungs was done with a 4% hydroxyethyl starch (HES 200/0.5, Serumwerk Bernburg) containing perfusion buffer through the pulmonary artery (1 ml/min). Lung function parameters were recorded automatically and resistance measured by HSE-HA PulmodynW Software (Harvard Apparatus). After a 20-minute equilibration period, lungs were perfused with increasing concentrations of MCh (0.1 μM, 1 μM, 10 μM, and 100 μM) for 10 minutes each, separated by a washout period (10 min) with buffer. For graphical and statistical analysis, the mean resistance values were calculated from the last ten time stamps of each 5-min period.

### Histopathology

Whole lung tissue sections were fixed in Histofix (4.5%, Carl Roth) overnight and embedded in paraffin. Slices (4 μm) were stained with either H&E or PAS and examined using a Zeiss Axio Imager.M2 with the AxioCam MRc camera. Tissue inflammation and infiltration was evaluated on H&E stained sections. PAS-positive goblet cells were quantified in percent of counted cells which have been determined by visible nuclei.

### Statistics

Statistical significance of differences was defined as a *P*<0.05 and denoted with asterisks: *0.05, **0.01 and ***0.001. All calculations were performed using GraphPad Prism 6.0 (GraphPad Software) and SPSS Statistics Version 22 (IBM). If not stated otherwise, data were statistically analyzed using Kruskal-Wallis one-way-analyses and Mann-Whitney U tests.

## Results and Discussion

**Fig 1:**
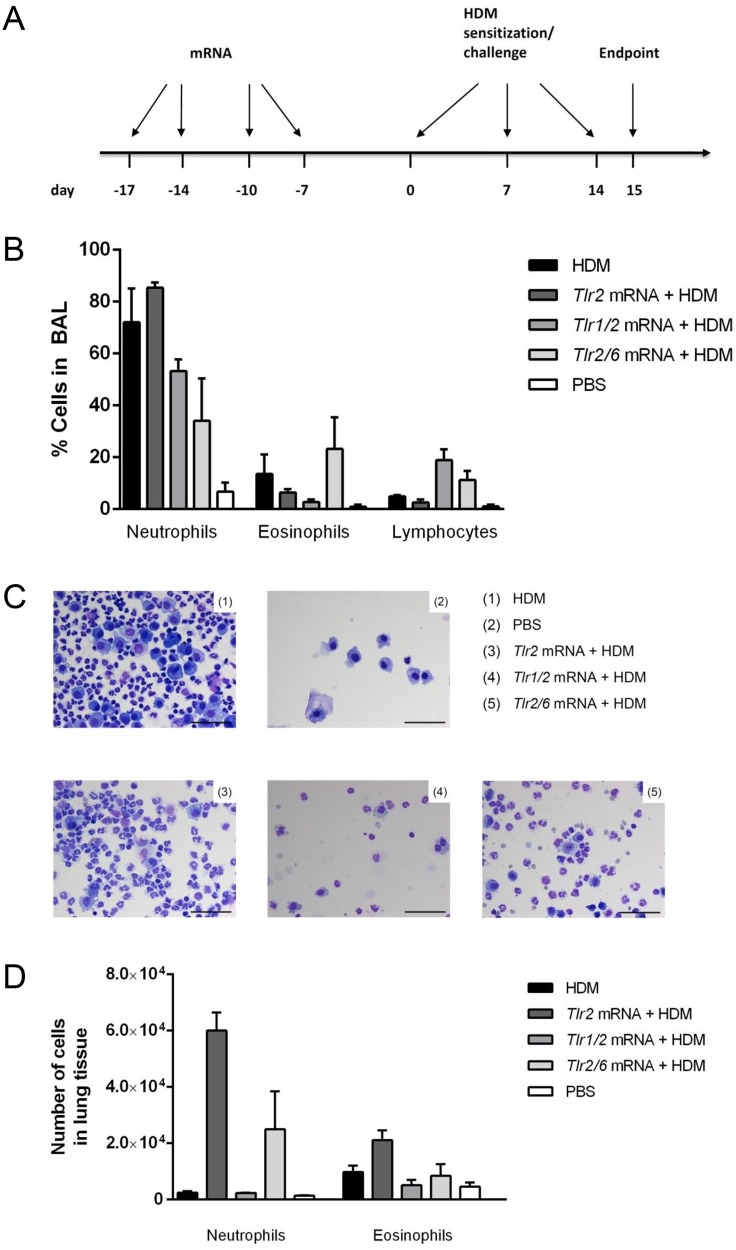
Inflammatory cells in BALF and lung tissue. (A) Mice were treated according to the injection schedule for the HDM-induced asthma model. (B) BALF was centrifuged and cells were analyzed via differential cell count. Differences remained non-significant. Data are presented as mean ± SEM; n = 3. (C) Representative micrographs of BALF cellspin preparations are shown (scale 100 μm, magnification x200). (D) Levels of neutrophils and eosinophils in lung tissue were measured via flow cytometry. Differences remained non-significant. Data are presented as mean ± SEM; n = 3.

Mice received combinations of *Tlr* mRNA (Table 1) at four determined time points (-17, -14, -10 and -7 days) before sensitization and challenge with HDM (Fig 1A). At day 15, the predetermined endpoint of the study, mice were sacrificed and analyzed.

**Table 1:** Groups of mice and their respective treatment following the injection schedule of Fig 1A.

Treatment (n = 9 per group)		Endpoint
Day -17, -14, -10, -7	Day 0, 7, 14	Day 15
*Tlr1/2* mRNA	HDM	Day 15
*Tlr2* mRNA	HDM	Day 15
*Tlr2/6* mRNA	HDM	Day 15
PBS	HDM	Day 15
PBS	PBS	Day 15

Mice were treated intratracheally with combinations of *Tlr* mRNA at day -17,-14, -10 and -7 prior to the first sensitization with House dust mite extract (HDM), indicated as day 0. Further intratracheal injections with HDM followed on day 7 and 14. On day 15, the predetermined endpoint of the study, mice were sacrificed and several readouts were performed. From each group n = 3 mice were subjected to BALF and FACS analysis, while IPL was performed on the remaining n = 6 mice.

Bronchial alveolar lavage fluid (BALF) cells were obtained at the time of sacrifice and analyzed via differential cell count (Fig 1B). Here, delivery of *Tlr1/2* mRNA led to decreased levels of neutrophils and eosinophils in BALF, when compared to untreated controls. Treatment with *Tlr2/6* mRNA resulted in higher amounts of eosinophils but still reduced neutrophilic inflammation. Levels of neutrophils were increased after the administration of *Tlr2* mRNA, eosinophils and lymphocytes were slightly diminished. Representative micrographs shown in Fig 1C additionally illustrate the decline of inflammatory cells after administration of *Tlr1/2* mRNA when compared to the HDM group.

In order to investigate the local effect of *Tlr* mRNA application on immune cells in lung tissue, we isolated and stained lung cells and subjected them to FACS analysis (Fig 1D). Similar to observed results in the BALF, delivery of *Tlr1/*2 mRNA was able to reduce the number of eosinophils in lung tissue. *Tlr2* mRNA led to a rise of eosinophils and furthermore markedly increased levels of neutrophils. Also treatment with *Tlr2/6* mRNA resulted in higher levels of neutrophils and did not reduce eosinophilic inflammation in lung tissue.

**Fig 2:**
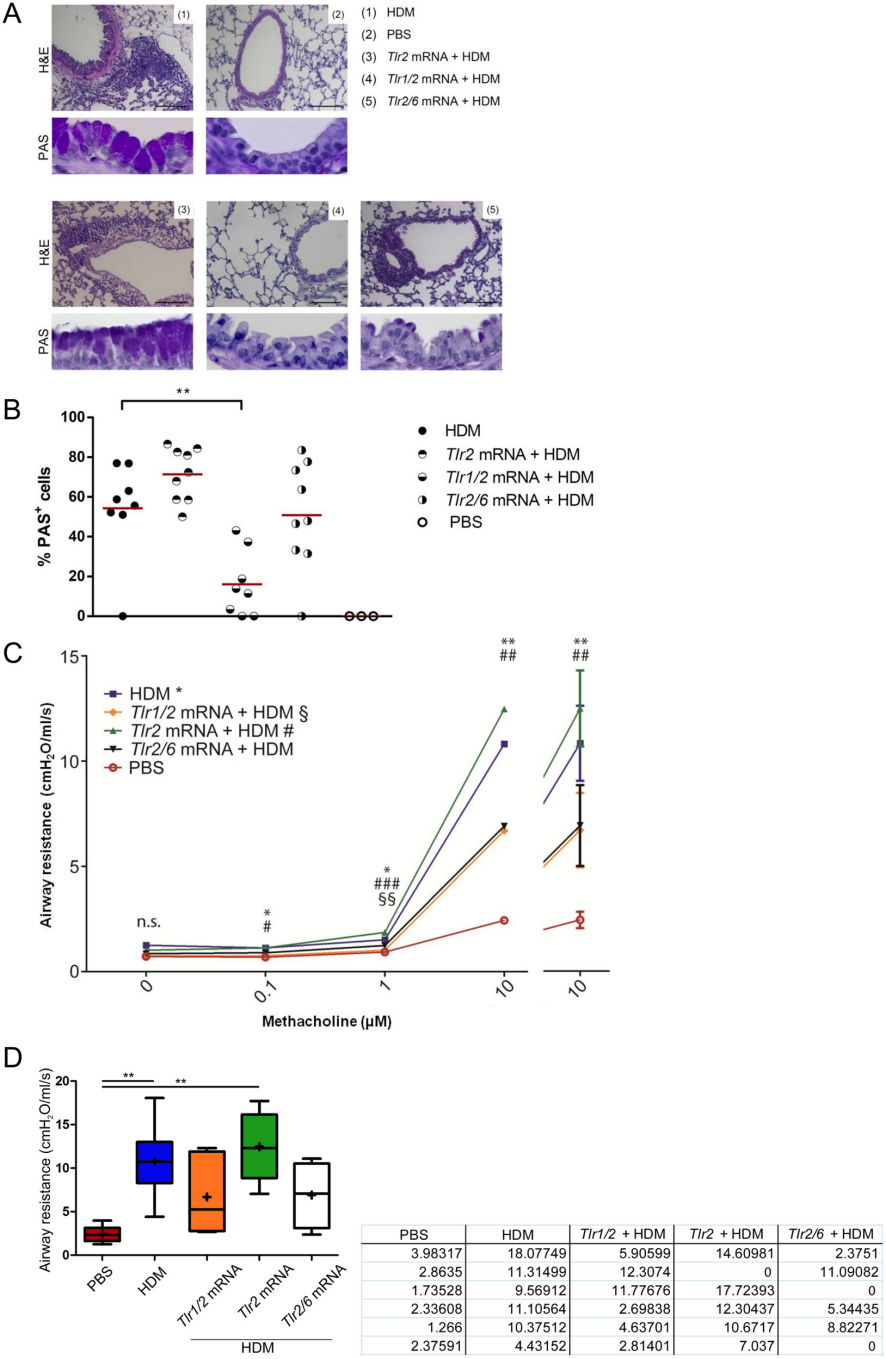
Tissue reaction and lung function after *Tlr* mRNA treatment. (A) Tissue inflammation and goblet cell metaplasia were analyzed on H&E- and PAS-stained lung sections. Representative micrographs are shown (original magnification of H&E sections: x200, scale 100 μm, PAS sections: x400). (B) PAS stained lung sections were analyzed to quantify PAS^+^ cells. Data are represented as individual mice, horizontal lines state means; n = 9. (C) Airway resistance was measured in response to rising concentrations of methacholine (MCh) using the isolated, perfused and ventilated lung (IPL). Statistical analysis was separately performed for each MCh concentration, * and # are PBS vs. the respective group, § is *Tlr1/2* vs. *Tlr2*. *P ≤ 0.05, **P ≤ 0.01, and ***P ≤ 0.001. Statistical results for each concentration can be found in S1 Table. (D) Data at 10 μM MCh are presented as Box and Whiskers blot. Medians are shown as +. Tukey’s Multiple Comparison Test **P < 0.01. Data are represented as means; data at 10 μM MCh are additionally stated as means ± SEM; n = 6 (*Tlr2* n = 5, *Tlr2/6* n = 4). Tabular values at 10 μM MCh (cmH2O/ml/s), values stated as “0” were excluded due to damage of the lungs.

In lung sections, either stained with H&E or PAS, we observed markedly reduced peribronchial, perivascular and interstitial tissue inflammation in lungs of mice treated with *Tlr1/2* mRNA (Fig 2A). Delivery of *Tlr1/2* mRNA was furthermore associated with a significant reduction of goblet cells in airways (*P* = 0.007) (Fig 2B). Administration of *Tlr2* mRNA did not dampen lung inflammation (Fig 2A) and resulted in a rather higher degree of goblet cell metaplasia when compared to untreated HDM controls (Fig 2B). Lung tissue inflammation was slightly reduced after treatment with *Tlr2/6* mRNA, whereas no differences in goblet cell metaplasia could be detected.

Next, we wanted to determine whether the administration of combinations of chemically modified *Tlr1*, *2* and *6* mRNA modulates airway hyperresponsiveness of mice in HDM-induced asthma. We therefore measured lung function in terms of airway resistance by using the *ex vivo* model of the isolated, perfused and ventilated lung (IPL).

We observed considerably reduced airway resistance values following *ex vivo* methacholine (MCh) challenge after delivery of *Tlr1/2* mRNA prior to HDM administration (Fig 2C). Likewise, application of *Tlr2/6* mRNA was associated with diminished airway resistance, indicating decreased airway hyperresponsiveness in these two groups. However, delivery of *Tlr2* mRNA led to slightly higher resistance values than in untreated asthmatic controls and thus to a decline in lung function.

In conclusion, we observed that intratracheal administration of *Tlr1/2* and *Tlr2/6* mRNA resulted in markedly reduced lung inflammation. *Tlr1/2* mRNA application led to a concomitant improved lung function *in vivo*. Administration of *Tlr2* mRNA alone showed no improvement compared to untreated HDM controls.

Whereas some aspects, such as the role of TLR4 [23–26], in asthma pathogenesis have been studied elaborately, little is known about TLR1/2 and TLR2/6 heterodimers in this context and some studies reveal contrary results [27–30].

As mentioned before, previous studies presented protective effects of TLR1 and 6 on atopic asthma in humans [21,31]. In line with these findings, we observed that treatment with the combination of *Tlr1/2* -and to some extent *Tlr2/6* mRNA- tended to result in a better asthma outcome *in vivo*. Percentages of neutrophils and eosinophils in BALF were reduced after *Tlr1/2* administration when compared to the HDM control group, also absolute numbers of eosinophils in lung tissue were diminished. Furthermore, these mice showed notably improved lung function and significantly reduced pulmonary mucus production.

However, these findings did not hold true for treatment with *Tlr2* mRNA alone. In contrast, *Tlr2* mRNA treatment promotes a rather proinflammatory phenotype regarding not only BALF and lung tissue but also lung function and histological analyses. These observations are in line with reports describing a crucial role of TLR2 being overexpressed in fatal asthma patients [32] and in patients suffering from persistent allergic rhinitis [33]. In a study investigating an ovalbumin-induced mouse model of asthma, the allergic response appeared to be largely TLR2 dependent, with significantly reduced allergic immune responses in TLR2-deficient mice [34]. The specific interaction of HDM components with TLR2, promoting a Th2 biased allergic immune response and close cross-talk between receptor pathways might serve as another explanation for this observation [35].

## Conclusions

Allergic asthma is a major burden worldwide and although several therapeutic options are available to treat asthma symptoms, especially cases of severe asthma are still difficult to control [36–38]. To target individual patient’s needs and provide tailored treatment, clinical approaches in the field of immunotherapy to target TLRs and Th responses are already subject of current research [39]. Due to the rather small number of mice, the present data should be interpreted in the context of the experimental design and considered as a pilot-study for future research. In this regard, analysis of TLR1/2 and/or TLR2/6 overexpressing mice would be of special interest. Despite some limitations of the study, our data point to a potentially protective effect of *Tlr1/2* mRNA treatment on HDM induced asthma. New insights into the role of TLRs in atopic asthma combined with novel therapeutic tools, such as cmRNA transcripts, can be considered promising targets of future research in the field of asthma management and prevention.

## Supporting Information

S1 Table Tabular statistical results obtained by performing the one-way ANOVA with Tukey’s Multiple Comparison Test as post test for comparison of individual groups at each MCh concentration. *P < 0.05; **P < 0.01 and ***P < 0.001.
(EPS)

## References

[1] Braun-Fahrländer C, Riedler J, Herz U, Eder W, Waser M, Grize L, et al Environmental exposure to endotoxin and its relation to asthma in school-age children. N Engl J Med. 2002;347: 869–77. 10.1056/NEJMoa020057 12239255

[2] Strachan DP. Hay fever, hygiene, and household size. BMJ. 1989;299: 1259–60. Available: http://www.ncbi.nlm.nih.gov/pubmed/2513902. 2513902 10.1136/bmj.299.6710.1259 PMC1838109

[3] Martinez FD, Holt PG. Role of microbial burden in aetiology of allergy and asthma. Lancet (London, England). 1999;354 Suppl: SII12–5. Available: http://www.ncbi.nlm.nih.gov/pubmed/10507253. 10.1016/s0140-6736(99)90437-3 10507253

[4] Matricardi PM, Rosmini F, Riondino S, Fortini M, Ferrigno L, Rapicetta M, et al Exposure to foodborne and orofecal microbes versus airborne viruses in relation to atopy and allergic asthma: epidemiological study. BMJ. 2000;320: 412–7. Available: http://www.ncbi.nlm.nih.gov/pubmed/10669445. 10669445 10.1136/bmj.320.7232.412 PMC27285

[5] Brown EM, Arrieta M-C, Finlay BB. A fresh look at the hygiene hypothesis: how intestinal microbial exposure drives immune effector responses in atopic disease. Semin Immunol. 2013;25: 378–87. 10.1016/j.smim.2013.09.003 24209708

[6] Sherriff A, Golding J, Alspac Study Team. Hygiene levels in a contemporary population cohort are associated with wheezing and atopic eczema in preschool infants. Arch Dis Child. 2002;87: 26–9. Available: http://www.ncbi.nlm.nih.gov/pubmed/12089117. 12089117 10.1136/adc.87.1.26 PMC1751124

[7] Akira S, Takeda K, Kaisho T. Toll-like receptors: critical proteins linking innate and acquired immunity. Nat Immunol. 2001;2: 675–80. 10.1038/90609 11477402

[8] Elias JA, Lee CG, Zheng T, Ma B, Homer RJ, Zhu Z. New insights into the pathogenesis of asthma. J Clin Invest. 2003;111: 291–7. 10.1172/JCI17748 12569150 PMC151878

[9] Kuperman D, Schofield B, Wills-Karp M, Grusby MJ. Signal transducer and activator of transcription factor 6 (Stat6)-deficient mice are protected from antigen-induced airway hyperresponsiveness and mucus production. J Exp Med. 1998;187: 939–48. Available: http://www.ncbi.nlm.nih.gov/pubmed/9500796. 9500796 10.1084/jem.187.6.939 PMC2212182

[10] Robinson DS, Hamid Q, Ying S, Tsicopoulos A, Barkans J, Bentley AM, et al Predominant TH2-like bronchoalveolar T-lymphocyte population in atopic asthma. N Engl J Med. 1992;326: 298–304. 10.1056/NEJM199201303260504 1530827

[11] Gonzalo JA, Lloyd CM, Kremer L, Finger E, Martinez-A C, Siegelman MH, et al Eosinophil recruitment to the lung in a murine model of allergic inflammation. The role of T cells, chemokines, and adhesion receptors. J Clin Invest. 1996;98: 2332–45. 10.1172/JCI119045 8941651 PMC507684

[12] Cohn L, Tepper JS, Bottomly K. IL-4-independent induction of airway hyperresponsiveness by Th2, but not Th1, cells. J Immunol. 1998;161: 3813–6. Available: http://www.jimmunol.org/content/161/8/3813.full. 9780144

[13] Bousquet J, Michel F-B. International consensus report on diagnosis and management of asthma. Allergy. 1992;47: 129–32. 10.1111/j.1398-9995.1992.tb00952.x 1514662

[14] Sears MR. The definition and diagnosis of asthma. Allergy. 1993;48: 12–6; discussion 22–3. Available: http://www.ncbi.nlm.nih.gov/pubmed/8109702. 10.1111/j.1398-9995.1993.tb04692.x 8109702

[15] Karikó K, Muramatsu H, Welsh FA, Ludwig J, Kato H, Akira S, et al Incorporation of pseudouridine into mRNA yields superior nonimmunogenic vector with increased translational capacity and biological stability. Mol Ther. 2008;16: 1833–40. 10.1038/mt.2008.200 18797453 PMC2775451

[16] Karikó K, Muramatsu H, Ludwig J, Weissman D. Generating the optimal mRNA for therapy: HPLC purification eliminates immune activation and improves translation of nucleoside-modified, protein-encoding mRNA. Nucleic Acids Res. 2011;39: e142 10.1093/nar/gkr695 21890902 PMC3241667

[17] Kormann MSD, Hasenpusch G, Aneja MK, Nica G, Flemmer AW, Herber-Jonat S, et al Expression of therapeutic proteins after delivery of chemically modified mRNA in mice. Nat Biotechnol. 2011;29: 154–7. 10.1038/nbt.1733 21217696

[18] Karikó K, Muramatsu H, Keller JM, Weissman D. Increased erythropoiesis in mice injected with submicrogram quantities of pseudouridine-containing mRNA encoding erythropoietin. Mol Ther. 2012;20: 948–53. 10.1038/mt.2012.7 22334017 PMC3345990

[19] Mays LE, Ammon-Treiber S, Mothes B, Alkhaled M, Rottenberger J, Müller-Hermelink ES, et al Modified Foxp3 mRNA protects against asthma through an IL-10-dependent mechanism. J Clin Invest. 2013;123: 1216–28. 10.1172/JCI65351 23391720 PMC3582134

[20] Mahiny AJ, Dewerth A, Mays LE, Alkhaled M, Mothes B, Malaeksefat E, et al In vivo genome editing using nuclease-encoding mRNA corrects SP-B deficiency. Nat Biotechnol. 2015;33: 584–6. 10.1038/nbt.3241 25985262

[21] Kormann MSD, Depner M, Hartl D, Klopp N, Illig T, Adamski J, et al Toll-like receptor heterodimer variants protect from childhood asthma. J Allergy Clin Immunol. 2008;122: 86–92, 92.e1–8. 10.1016/j.jaci.2008.04.039 18547625

[22] Moreira AP, Cavassani KA, Ismailoglu UB, Hullinger R, Dunleavy MP, Knight DA, et al The protective role of TLR6 in a mouse model of asthma is mediated by IL-23 and IL-17A. J Clin Invest. 2011;121: 4420–32. 10.1172/JCI44999 22005301 PMC3204826

[23] Chen S. Association between the TLR4 +896A>G (Asp299Gly) polymorphism and asthma: a systematic review and meta-analysis. J Asthma. 2012;49: 999–1003. 10.3109/02770903.2012.738270 23574398

[24] Sahin F, Yıldız P, Kuskucu A, Kuskucu MA, Karaca N, Midilli K. The effect of CD14 and TLR4 gene polymorphisms on asthma phenotypes in adult Turkish asthma patients: a genetic study. BMC Pulm Med. 2014;14: 20 10.1186/1471-2466-14-20 24524443 PMC3928321

[25] Kim DH, Choi E, Lee J-S, Lee NR, Baek SY, Gu A, et al House Dust Mite Allergen Regulates Constitutive Apoptosis of Normal and Asthmatic Neutrophils via Toll-Like Receptor 4. Fessler MB, editor. PLoS One. 2015;10: e0125983 10.1371/journal.pone.0125983 25973752 PMC4431853

[26] Fagerås Böttcher M, Hmani-Aifa M, Lindström A, Jenmalm MC, Mai X-M, Nilsson L, et al A TLR4 polymorphism is associated with asthma and reduced lipopolysaccharide-induced interleukin-12(p70) responses in Swedish children. J Allergy Clin Immunol. 2004;114: 561–7. 10.1016/j.jaci.2004.04.050 15356557

[27] Hoffjan S, Stemmler S, Parwez Q, Petrasch-Parwez E, Arinir U, Rohde G, et al Evaluation of the toll-like receptor 6 Ser249Pro polymorphism in patients with asthma, atopic dermatitis and chronic obstructive pulmonary disease. BMC Med Genet. 2005;6: 34 10.1186/1471-2350-6-34 16188043 PMC1262722

[28] Bezemer GFG, Sagar S, van Bergenhenegouwen J, Georgiou NA, Garssen J, Kraneveld AD, et al Dual role of Toll-like receptors in asthma and chronic obstructive pulmonary disease. Pharmacol Rev. 2012;64: 337–58. 10.1124/pr.111.004622 22407613

[29] Koponen P, Vuononvirta J, Nuolivirta K, Helminen M, He Q, Korppi M. The association of genetic variants in toll-like receptor 2 subfamily with allergy and asthma after hospitalization for bronchiolitis in infancy. Pediatr Infect Dis J. 2014;33: 463–6. 10.1097/INF.0000000000000253 24445834

[30] Månsson Kvarnhammar A, Tengroth L, Adner M, Cardell L-O. Innate Immune Receptors in Human Airway Smooth Muscle Cells: Activation by TLR1/2, TLR3, TLR4, TLR7 and NOD1 Agonists. Proost P, editor. PLoS One. 2013;8: e68701 10.1371/journal.pone.0068701 23861935 PMC3701658

[31] Renkonen J, Joenväärä S, Parviainen V, Mattila P, Renkonen R. Network analysis of single nucleotide polymorphisms in asthma. J Asthma Allergy. 2010;3: 177–86. 10.2147/JAA.S14459 21437052 PMC3047920

[32] Ferreira DS, Annoni R, Silva LFF, Buttignol M, Santos ABG, Medeiros MCR, et al Toll-like receptors 2, 3 and 4 and thymic stromal lymphopoietin expression in fatal asthma. Clin Exp Allergy. 2012;42: 1459–71. 10.1111/j.1365-2222.2012.04047.x 22994343 PMC3459227

[33] Cui X-Y, Chen X, Yu C-J, Yang J, Lin Z-P, Yin M, et al Increased expression of toll-like receptors 2 and 4 and related cytokines in persistent allergic rhinitis. Otolaryngol Head Neck Surg. 2015;152: 233–8. 10.1177/0194599814562173 25505260

[34] Li X, Chen Q, Chu C, You H, Jin M, Zhao X, et al Ovalbumin-induced experimental allergic asthma is Toll-like receptor 2 dependent. Allergy Asthma Proc. 2014;35: e15–20. 10.2500/aap.2014.35.3735 24717780

[35] Liu C-F, Drocourt D, Puzo G, Wang J-Y, Riviere M. Innate immune response of alveolar macrophage to house dust mite allergen is mediated through TLR2/-4 co-activation. PLoS One. 2013;8: e75983 10.1371/journal.pone.0075983 24098413 PMC3787959

[36] Guilbert TW, Bacharier LB, Fitzpatrick AM. Severe asthma in children. J allergy Clin Immunol Pract. 2014;2: 489–500. 10.1016/j.jaip.2014.06.022 25213041 PMC4589165

[37] Fleming L, Murray C, Bansal AT, Hashimoto S, Bisgaard H, Bush A, et al The burden of severe asthma in childhood and adolescence: results from the paediatric U-BIOPRED cohorts. Eur Respir J. 2015;46: 1322–33. 10.1183/13993003.00780-2015 26405287

[38] Holgate ST, Polosa R. The mechanisms, diagnosis, and management of severe asthma in adults. Lancet (London, England). 2006;368: 780–93. 10.1016/S0140-6736(06)69288-X 16935689

[39] Aryan Z, Rezaei N. Toll-like receptors as targets for allergen immunotherapy. Curr Opin Allergy Clin Immunol. 2015;15: 568–74. 10.1097/ACI.0000000000000212 26418475

