# Correction: mRNA-Mediated Gene Supplementation of Toll-Like Receptors as Treatment Strategy for Asthma *In Vivo*

**DOI:** 10.1371/journal.pone.0197705

**Published:** 2018-05-15

**Authors:** Franziska Zeyer, Benedikt Mothes, Clara Will, Melanie Carevic, Jennifer Rottenberger, Bernd Nürnberg, Dominik Hartl, Rupert Handgretinger, Sandra Beer-Hammer, Michael S. D. Kormann

The following information is missing from the Funding section: This study was supported by a European Research Council (ERC) grant (no: 637752).
